# Markers of compromised gut epithelial barrier integrity increase during the menopause transition

**DOI:** 10.1172/JCI205059

**Published:** 2026-07-09

**Authors:** Albert Shieh, Marta Epeldegui, Arun S. Karlamangla, Rheinallt Jones, Roberto Pacifici, Gail A. Greendale

**Affiliations:** 1 Division of Geriatrics, Department of Medicine, and; 2 Department of Microbiology, Immunology & Molecular Genetics, David Geffen School of Medicine at UCLA, Los Angeles, California, USA.; 3 Department of Pediatrics and; 4 Division of Endocrinology, Department of Medicine, Emory University, Atlanta, Georgia, USA.

**Keywords:** Endocrinology, Inflammation, Reproductive biology, Epidemiology, Population health research

## Abstract

**BACKGROUND:**

In female murine models, one source of inflammation is a menopause-related increase in gut permeability. We examined whether the menopause transition (MT) in women is associated with an increase in markers of gut epithelial dysfunction and gut microbial product translocation, signals of compromised gut epithelial barrier integrity.

**METHODS:**

In 964 women, we measured markers of gut epithelial dysfunction (fatty acid binding protein 2, FABP2) and gut microbial antigen translocation (soluble CD14, sCD14) using sera collected before, during, and after the MT. Multivariable mixed effects regressions fit piecewise linear models to repeated FABP2 or sCD14 measures relative to time from final menstrual period (FMP). Covariates were age at FMP, race and ethnicity, and BMI.

**RESULTS:**

FABP2 and sCD14 did not change significantly until 2.5 years pre-FMP. At that point, FABP2 began rising; sCD14 began increasing 6 months later. FABP2 and sCD14 peaked 6 and 6.5 years after FMP, respectively; subsequent levels remained stable. During the ~9-year interval of MT-related gain in gut barrier compromise markers, annual FABP2 and sCD14 increases were 2.6% (95% CI: 1.7% to 3.4%) and 0.8% (95% CI: 0.6% to 1.1%), respectively, among White women with sample-average BMI and age at FMP. FABP2 and sCD14 change rates did not differ significantly by race and ethnicity, BMI, or age at FMP.

**CONCLUSION:**

The MT is associated with a rise in markers of compromised gut barrier integrity, suggesting that this pathway of inflammation, previously described in animal models, occurs in humans.

**FUNDING:**

NIH U01NR004061, U01AG012505, U01AG012535, U01AG012531, U01AG012539, U01AG012546, U01AG012553, U01AG012554, U01AG012495, U19AG063720, 5R01AR081794, R01AR075729.

## Abstract 2

The study demonstrates that gut epithelilal damage and activation of the immune system increase rapidly during the menopause transition.

## Introduction

Inflammation contributes to the pathogenesis of numerous conditions that commonly affect older adults, including osteoporosis, diabetes mellitus, cardiovascular disease, and dementia (1–6). A newly uncovered mechanism of inflammation in female murine models is a menopause-related increase in gut permeability (7–16). Specifically, murine experimental models demonstrated that depletion of sex steroid hormones results in diminished abundance of gut epithelial junction proteins and increased gut epithelial permeability (7, 8, 13–16). This, in turn, permits the translocation of gut microbial antigens from the intestinal lumen into the subepithelial compartments, triggering aberrant immune cell activation and low-grade inflammation (9–12).

In the current study, we investigated whether the menopause transition (MT) contributes to increased intestinal epithelial permeability in humans. In a previously published pilot study of 65 participants in the Study of Women’s Health Across the Nation (SWAN), we measured circulating markers of compromised gut epithelial barrier integrity (characterized by gut barrier dysfunction and translocation of gut microbe–derived products) at 2 time points: once in premenopause and a second time in postmenopause. To assess gut barrier dysfunction, we measured fatty acid binding protein 2 (FABP2), a protein that is produced by enterocytes and released into the bloodstream when the gut mucosa is damaged (17). To index translocation of gut microbial antigenic material, we measured soluble CD14 (sCD14), which is secreted by immune cells in response to sensing lipopolysaccharide (LPS) (18–20). Indeed, we uncovered that both FABP2 and sCD14 increased significantly from pre- to postmenopause LPS (21, 22). While these findings indicate that markers of compromised gut epithelial barrier integrity increase when women become postmenopausal, they do not disaggregate whether this increase is driven by ovarian (menopause) versus chronological age, as both processes occur contemporaneously.

The primary objective of this study was to undertake a comprehensive investigation of whether the MT is associated with a heightened abundance of markers of compromised gut epithelial barrier integrity in a larger study sample. Specifically, we assayed FABP2 and sCD14 in stored plasma samples collected at multiple time points before, during, and after the MT from 964 SWAN participants who reached menopause at different ages. We then modeled repeated measures of each marker as function of ovarian age (years before or after the final menstrual period [FMP]). Using this construct, the FMP date equals time 0. Time before the FMP is negative, and time after FMP is positive; for instance, FMP –4 years means 4 years before, and FMP +4 years means 4 years after the FMP date. This FMP-based modeling strategy allows us to discern whether changes in an outcome (in this case, FABP2 or sCD14) result from ovarian versus chronological aging. If change in FABP2 or sCD14 were related to the MT, we would expect to see changes anchored to the FMP date. Based on observed patterns of change in other outcomes associated with ovarian aging (e.g., bone mineral density, body composition, cognition), we hypothesized that FABP2 and/or sCD14 would start to increase abruptly before the FMP, continue to rise at a high rate until several years after FMP, and decelerate thereafter (23–26). Our secondary objective was to examine whether race and ethnicity, BMI, and age at FMP are associated with rates of FABP2 and sCD14 change.

## Results

### Participant characteristics.

The study sample consisted of a subset of 964 women from the SWAN Bone Cohort (parent Bone Cohort *N* = 2,365). Study sample participants had a natural menopause and known FMP date and did not use sex steroid hormones or medications that could affect gut barrier integrity or inflammation (e.g., NSAIDs, glucocorticoids, and antibiotics). The racial/ethnic composition of our analysis sample was 263 Black (27%), 133 Chinese (14%), 147 Japanese (15%), and 421 White (44%). As shown in Table 1, characteristics of the current analysis sample and full Bone Cohort at the SWAN baseline visit were similar.

We assayed FABP2 and sCD14 from SWAN repository plasma samples. Henceforth, we refer to the initial analysis visit as the first visit at which study sample participants had FABP2 and sCD14 assessments. At the initial analysis visit, average age was 48.87 years, mean time from FMP was –2.79 years, and average BMI was 27.45 kg/m^2^. Also, at this time point, FABP2 values were not normally distributed, but sCD14 levels were. Median FABP2 and mean sCD14 values were 1,007 pg/mL and 740 ng/mL, respectively. Compared with White women, Black participants had similar FABP2 (*P* = 0.6), but lower sCD14 (*P* < 0.0001), values. Relative to those of White women, FABP2 levels were greater (*P* < 0.0001) and sCD14 values were lower (*P* < 0.0001) among Chinese participants. Japanese women had lower FABP2 (*P* < 0.0001) and sCD14 (*P* < 0.0001) levels than did White participants (Table 2).

Among the 964 analysis sample participants, we had 3,546 repeated assessments of FABP2 and sCD14. Plasma for these measurements was collected over a period spanning FMP –8.9 to FMP +16.3 years. Number of assessments, per participant, of compromised gut epithelial barrier integrity was median 4 [p25, p75 of 2, 5].

### Crude trajectories of FABP2 and sCD14 (LOESS plots).

**Figure 1:**
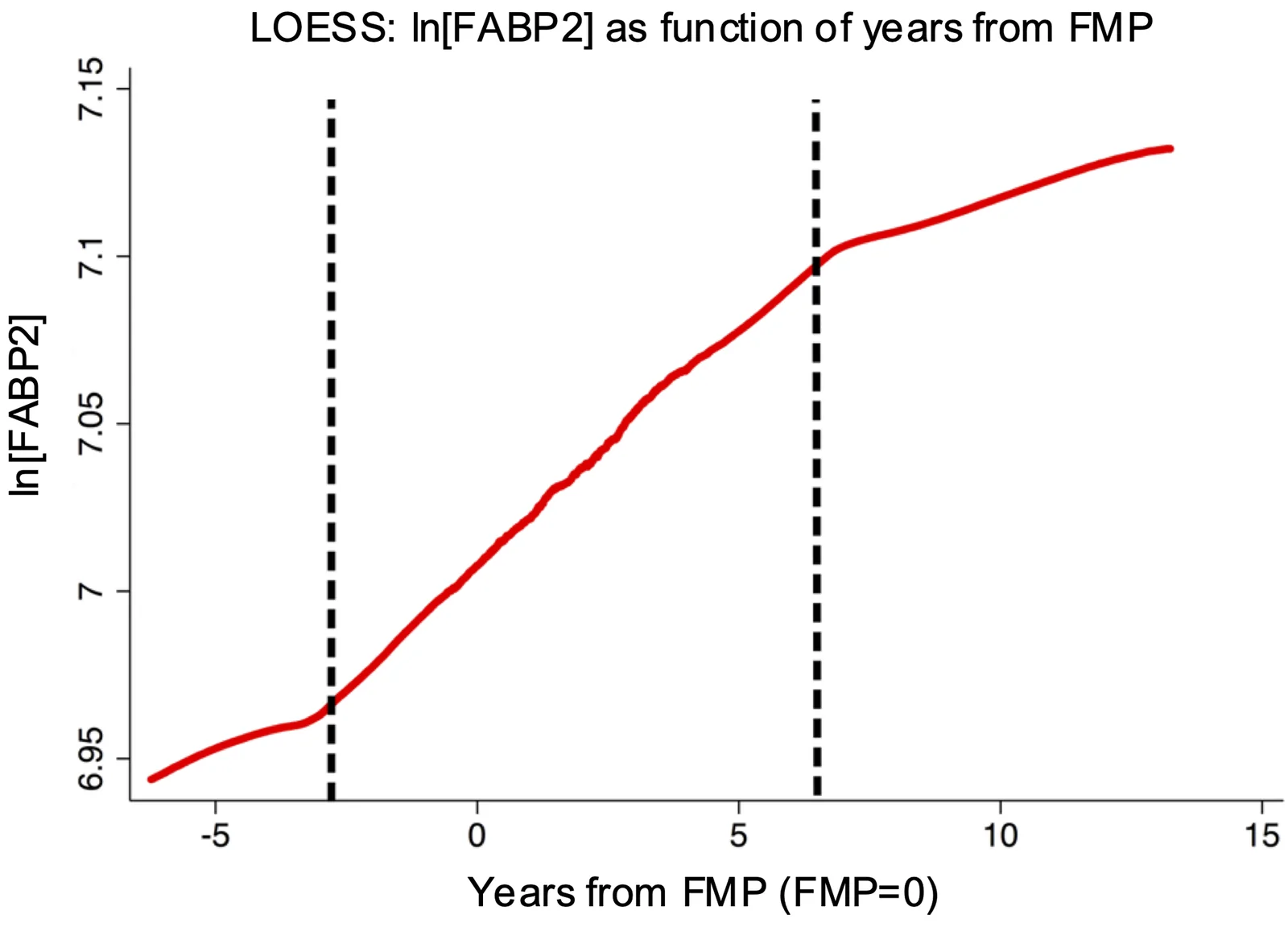
LOESS plot of ln[FABP2] in relation to number of years before or after the FMP. FABP2, fatty acid binding protein 2; FMP, final menstrual period.

**Figure 2:**
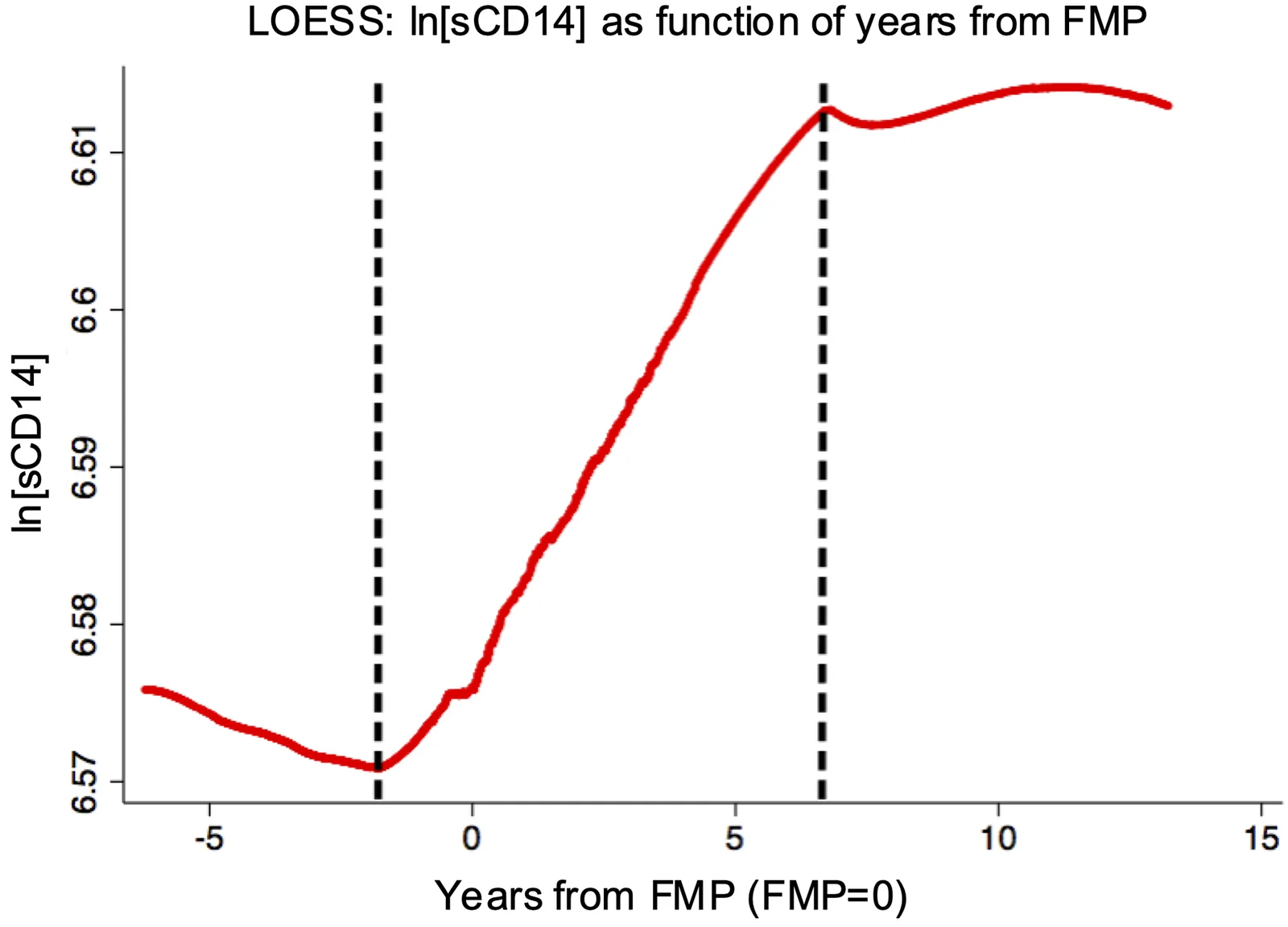
LOESS plot of ln[sCD14] in relation to number of years before or after the FMP. sCD14, soluble CD14; FMP, final menstrual period.

To visualize the crude, longitudinal pattern of change in each marker of compromised gut epithelial barrier integrity, we generated LOESS plots of natural log-transformed FABP2 (ln[FABP2], Figure 1) or sCD14 (ln[sCD14], Figure 2) as functions of years prior to and after the FMP. The trajectories of both markers were piecewise linear, consisting of 3 linear segments and 2 inflection points where the ln[FABP2] or ln[sCD14] slope changed from one segment to the next. Inflection points for ln[sCD14] trailed those of ln[FABP2] by 0.5 years. Specifically, the first inflection point occurred *before* the FMP for both markers: slopes for ln[FABP2] and ln[sCD14] increased (*P* < 0.0001 for slope change for each) 2.5 and 2 years pre-FMP, respectively. The second inflection point occurred *after* the FMP, with the ln[FABP2] and ln[sCD14] slopes decreasing at 6 years and 6.5 years post-FMP, respectively (*P* < 0.001 for slope change for each).

### Annual rates of change in FABP2 and the effects of race and ethnicity, BMI, and age at FMP.

To quantify annualized rate of change in ln[FABP2] (i.e., the ln[FABP2] slope), we used mixed effects linear regression to fit piecewise linear growth curves with 3 linear segments demarcated by the inflection points identified from the LOESS plots above (FMP –2.5 and FMP +6 years). We then multiplied ln[FABP2] slopes and 95% confidence intervals by 100 to allow interpretation as annualized percentage change in raw FABP2. We refer to the segment of the trajectory between the first and second inflection points as the period of MT-related increase in gut epithelial barrier compromise, and the periods before and after this period as pre- and postmenopause, respectively.

The first row of data in Table 3 shows the FABP2 slopes during premenopause, the period of MT-related increase in markers of compromised gut epithelial barrier integrity, and postmenopause; presented slopes are for the referent participant, defined as a White woman with sample-average values of age at FMP (52.16 years) and BMI (27.26 kg/m^2^). FABP2 did not change in premenopause (i.e., the slope was not statistically significantly different than 0; *P* = 0.2). During its period of MT-related increase (FMP –2.5 to FMP +6 years), FABP2 rose by 2.6% per year (95% CI 1.7 to 4.3%; *P* < 0.0001). In total, cumulative MT-related rise in FABP2 was 26.3%. Following its period of MT-related increase (postmenopause), FABP2 stabilized above premenopausal levels (the postmenopausal rate of change was not significantly different than 0, *P* = 0.4).

**Figure 3:**
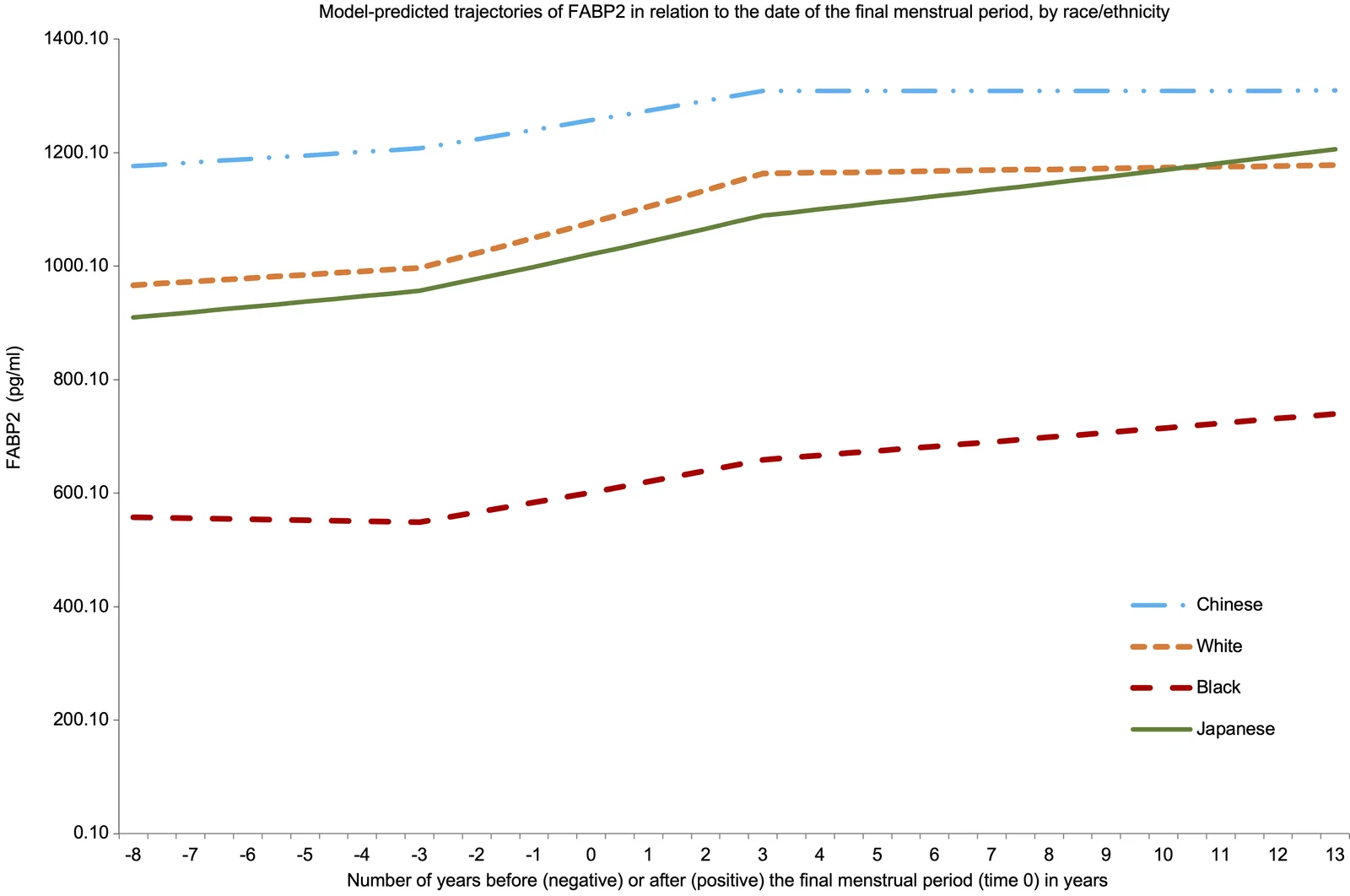
Model-predicted trajectories of FABP2 relative to the date of the FMP, by race and ethnicity. Trajectories estimated using mixed effects linear regression to fit repeated measures of FABP2 to years before or after the FMP, adjusting for race and ethnicity, BMI, and age at FMP. Values assume the sample mean values for age at FMP (52.16 years) and baseline BMI (27.26 kg/m^2^).

We also quantified the differences (relative to the reference sample) in percentage change per year of FABP2, by race and ethnicity, BMI, and age at FMP (Table 3). None of the reported slope differences were statistically significant, indicating that race and ethnicity, BMI, and age at FMP were not associated with rates of FABP2 change. To aid visualization of the data presented in Table 3, Figure 3 depicts the average race and ethnicity-specific trajectories of FABP2 as function of FMP time.

### Annual rates of change in sCD14 and the effects of race and ethnicity, BMI, and age at FMP.

Analogous to Table 3, Table 4 shows the sCD14 slopes (in percentage change per year) during premenopause, the period of MT-related increase, and postmenopause in the referent participant, plus differences (versus referent) in sCD14 change rates by race and ethnicity, BMI, and age at FMP.

**Figure 4:**
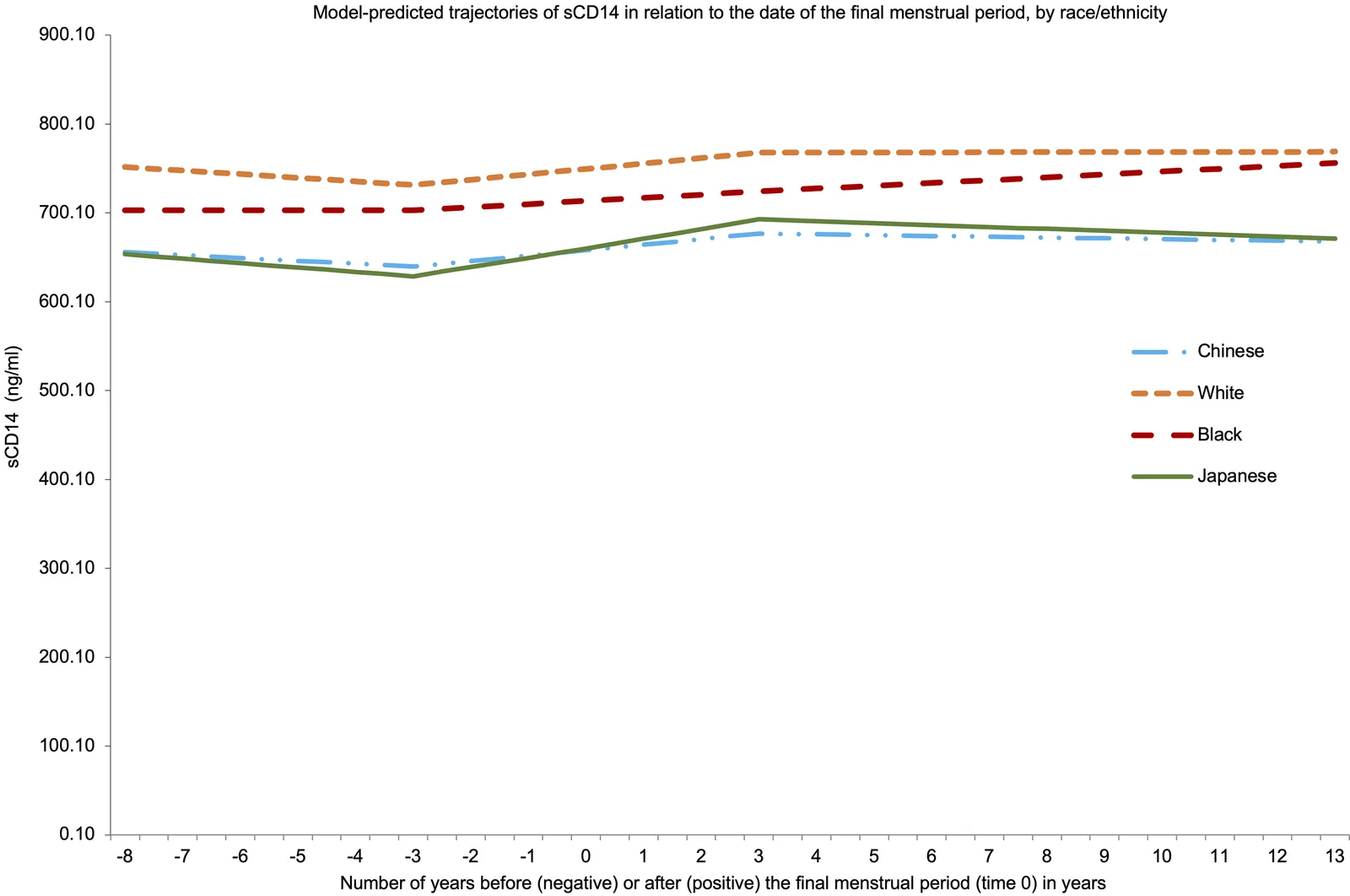
Model-predicted trajectories of sCD14 relative to the date of the FMP, by race and ethnicity. Trajectories estimated using mixed effects linear regression to fit repeated measures of sCD14 to years before or after the FMP, adjusting for race and ethnicity, BMI, and age at FMP. Values assume the sample mean values for age at FMP (52.16 years) and baseline BMI (27.26 kg/m^2^).

In the referent woman, the premenopausal sCD14 slope was not significantly different than 0 (*P* = 0.2). During its period of MT-related increase (FMP –2 to FMP +6.5 years), sCD14 grew by 0.8% annually (95% CI 0.6 to 1.1%; *P* < 0.0001). This amounts to a total MT-related gain of 7.5%. In postmenopause, rate of sCD14 change did not differ significantly from 0 (*P* = 0.4). As with FABP2, race and ethnicity, BMI, and age at FMP were not associated with rate of sCD14 change during premenopause, the period of MT-related increase, or postmenopause. Figure 4 presents the mean 3-piece sCD14 trajectories relative to FMP time by race and ethnicity.

### Gut barrier dysfunction as a pathway for gut microbial antigen translocation.

After substantiating an MT-related interval of FABP2 and sCD14 gain, we examined whether gut barrier dysfunction (indexed using FABP2) could be a pathway for translocation of gut microbial antigens (estimated using sCD14). First, we used individual fixed effects (IFE) regression to model the association of within-individual change in FABP2 (independent variable) with within-individual change in sCD14 (dependent variable) during the period spanning FMP –2.5 to FMP +6.5 years. Adjusted for BMI and chronological age, each 10% increase in FABP2 was associated with a 2.6% increase in sCD14 (95% confidence interval: 0.9%–4.7%; *P* < 0.01). Upon finding that greater FABP2 increase was associated with greater sCD14 increase, we added FABP2 to the model used to generate the sCD14 slopes presented above. Adjusting for FABP2 attenuated the rate of MT-related sCD14 gain by 13.8%.

## Discussion

This study characterized the longitudinal trajectories of markers of compromised gut epithelial barrier integrity (FABP2 and sCD14) from pre- to postmenopause. Before the MT (premenopause), FABP2 and sCD14 levels were stable. FABP2 and sCD14 levels began to rise 2.5 and 2 years before the FMP, respectively, marking the start of an MT-related increase in gut epithelial barrier compromise. FABP2 and sCD14 continued to rise at annual rates of 2.6% and 0.8%, respectively, with FABP2 peaking 6 years after FMP, and sCD14 reaching its high point 6 months later. Greater increase in FABP2 was associated with greater increase in sCD14. Following their periods of MT-related gain, FABP2 and sCD14 levels stabilized at their peak levels in postmenopause. Rates of FABP2 and sCD14 change were not associated with race and ethnicity, BMI, or age at FMP.

To our knowledge, the only currently published study to examine whether the MT is associated with compromised gut epithelial barrier integrity in generally healthy women was a pilot analysis of 65 SWAN participants in which we measured FABP2 and sCD14 once ~4 years before FMP and again ~4 years after FMP. During the intervening period, FABP2 and sCD14 rose by 22.8% and 8.9%, respectively (21). Although these results showed that markers of gut epithelial barrier compromise increase from before to after the FMP, they could not disentangle whether the rise is driven by ovarian versus chronological aging. The current analysis substantiates that this increase in markers of compromised gut epithelial barrier integrity is indeed associated with the MT. By referencing FABP2 and sCD14 against the number of years pre- or post-FMP, we uncovered an ~9-year interval of FABP2 and sCD14 gain that began abruptly ~2.5 years before the FMP and decelerated ~6.5 years after it. This pattern of change characterizes menopause-related outcomes such as bone mineral density, bone turnover markers, and body composition, although the precise timing of onset and offset of the MT-related acceleration varies by outcome (24–26).

Investigators can assess gut permeability using different approaches, but not all are practical in epidemiological studies (19). For instance, histologic and electron microscopic evaluations of biopsy samples directly assess gut epithelial cellular integrity and mucosal tight junctions (19, 27, 28), while enteral administration of nondigestible carbohydrates offers a functional assessment of gut permeability (19, 29, 30). In examinations of large community-based cohorts, these methodologies are not feasible: in such instances, circulating markers of compromised gut epithelial barrier integrity, characterized by gut barrier dysfunction and translocation of gut microbial products, can be assayed from blood samples (17–19, 31–33). We selected FABP2 as an indicator of gut barrier dysfunction because (a) it is produced by enterocytes and released into the bloodstream when the gut mucosa is damaged (17, 34); (b) its levels correlate with gut permeability, assessed by urinary excretion of orally ingested lactulose or rhamnose (35, 36); and (c) greater FABP2 levels correlate with greater levels of LPS (37). We utilized sCD14 as a marker of gut microbial product translocation because it is produced primarily by monocytes and macrophages in the presence of LPS (a cell wall component of Gram-negative bacteria, the majority of which resides in the gut) (18–20).

Most human studies using FABP2 and sCD14 as markers of compromised gut epithelial barrier integrity focused on individuals with pathologic conditions known to be associated with greater gut permeability (e.g., HIV, celiac disease, inflammatory bowel disease) (22, 38–52). As expected, FABP2 and sCD14 levels in such populations were qualitatively greater than those in our sample of generally healthy women. For instance, among individuals with HIV, median reported FABP2 and sCD14 levels ranged from 1,010 to 1,594 pg/mL and 1,529 to 2,121 ng/mL, respectively (22, 48). In contrast, median FABP2 and sCD14 levels in the current sample of SWAN participants were 1,007 pg/mL and 753 ng/mL, respectively. Our finding that the MT is associated with significant increases in FABP2 and sCD14 also indicates that these markers are sensitive enough to detect physiologic, and not just pathologic, diminutions of gut barrier integrity. Before these assays can be considered for clinical use, however, their reliability should be assessed using statistical measures such as the intraclass correlation coefficient. We could not compute the FABP2 and sCD14 intraclass correlation coefficients because we did not have enough participants with multiple biomarker assessments during time intervals when FABP2 and sCD14 levels were stable (either before FMP –2.5 years or after FMP +6.5 years).

Zonulin (a regulator of gut epithelial tight junctions) and lipopolysaccharide binding protein (LBP) are alternative indicators of gut barrier dysfunction and gut microbial product translocation, respectively (21, 53–57). We did not include zonulin in this study for 2 reasons. First, the magnitude of the correlation between zonulin levels and functional assessments of gut permeability (correlation coefficient < 0.2) (53, 54) is less than that for FABP2 (correlation coefficient 0.4 to 0.5) (35, 36, 53, 54). Second, some investigators have questioned whether the commercially available zonulin assay actually measures the stated analyte (58). We did not quantify LBP for this analysis because both sCD14 and LBP are produced in response to LPS (albeit via different mechanisms), and sCD14 levels were more strongly associated with estradiol levels during the MT in our pilot study (21).

The clinical implication of our results is that an MT-related acceleration in gut epithelial barrier dysfunction and subsequent immune activation likely occurs in women and that this could be a target for interventions to prevent its sequelae and associated medical conditions. Moreover, because rates of FABP2 and sCD14 change did not differ by race and ethnicity, an MT-associated loss of gut barrier integrity could be a therapeutic target in all women. Using bone as an example, preclinical studies have shown that some probiotic strains (7, 59, 60) or mixtures (7, 61–63) that upregulate intestinal junction protein expression reduce gut leak (7, 61, 63), prevent inflammation (7, 59–63), and mitigate or completely block menopause-related bone loss (7, 59, 60, 62, 63). Future research will examine whether changes in FABP2 and sCD14 are associated with changes in outcomes linked to menopause and inflammation (e.g., bone mineral density, bone turnover, insulin resistance) (6). If compromised gut barrier integrity proves to be a pathway of unfavorable health outcomes, clinical trials could test whether it is possible to prevent an MT-related loss of gut barrier integrity in humans.

Strengths of this study include repeated assessments of markers of compromised gut epithelial barrier integrity from pre- to postmenopause and a longitudinal FMP-based modeling strategy, which allowed us to discern the timing and magnitude of MT-related increases in FABP2 and sCD14.

The principal weakness of this analysis is that we did not directly assess gut permeability using histologic or functional approaches, as these methodologies were impractical for our cohort study. Rather, we selected circulating markers that indirectly reflect 2 physiologic characteristics of gut epithelial barrier compromise: gut epithelial damage (FABP2) and the subsequent translocation of Gram-negative bacterial antigens (sCD14). Our results support that these markers are sufficiently sensitive and precise to detect longitudinal alterations in gut barrier integrity in generally healthy women. A limitation of using sCD14 is that it is not specific for gut microbial translocation because it is produced by immune cells in response to LPS produced by any Gram-negative bacteria (18, 19, 48). Nonetheless, gains in sCD14 during the MT plausibly reflect a rise in gut microbial product translocation because the gut microbiota is the principal reservoir of Gram-negative microbes (20). Directly quantifying LPS levels was not feasible because our previously collected repository samples were subject to ubiquitous bacterial contamination, as not all collection and processing occurred in completely bacteria-free conditions (even commercially available, “sterile” collection tubes can be contaminated) (64). A final limitation is that SWAN did not collect data on use of probiotics or presence of all comorbidities that could impact gut epithelial barrier integrity. This limitation does not alter our conclusions, however, because (a) MT stage is not associated with the occurrence of conditions that commonly disrupt gut mucosal function (e.g., inflammatory bowel disease) (65); and (b) not being able to adjust for these conditions in analyses would have obscured the relationship of reproductive age with change in gut barrier integrity and reduced our ability to discern an association of time from FMP with FABP2 or sCD14.

In summary, to our knowledge, this is the first longitudinal human study to demonstrate an MT-related rise in markers of gut epithelial dysfunction and gut microbial antigen translocation in generally health women. Future research will interrogate the clinical significance of this increase in indicators of gut epithelial barrier compromise by testing whether changes in FABP2 and sCD14 are associated with changes in markers of immune activation and outcomes affected by menopause and inflammation.

## Methods

### Sex as a biological variable.

Only women were included in this study, as this analysis examined whether the MT is associated with an increase in signals of compromised gut epithelial barrier integrity.

### Description of the SWAN cohort.

SWAN is an ongoing, multicenter, longitudinal cohort study of the MT and aging. At the SWAN baseline visit, participants were 42 to 52 years old, had ≥1 ovary and an intact uterus, and were pre- (no change from usual menstrual bleeding) or early perimenopausal (less predictable menses at least once every 3 months). Women were recruited at 7 clinical sites. All sites recruited White women plus participants from one other racial/ethnic group. Boston, Chicago, Detroit, and Pittsburgh recruited Black women; Oakland enrolled Chinese participants; Newark recruited non-White Hispanic women; and Los Angeles enrolled Japanese participants. All racial/ethnic classifications were based on self-report.

The full SWAN cohort consists of 3,302 women. Since study inception in 1996, 1 baseline and 16 follow-up visits have occurred at a median (p25, p75) of 1.1 (1.0, 1.4) years between consecutive visits. Sixty-three percent of surviving women (*N* = 2,067) participated in the most recent follow-up visit, which was conducted between 2015 and 2018. By that time point, all women were postmenopausal.

### Study sample.

**Figure 5:**
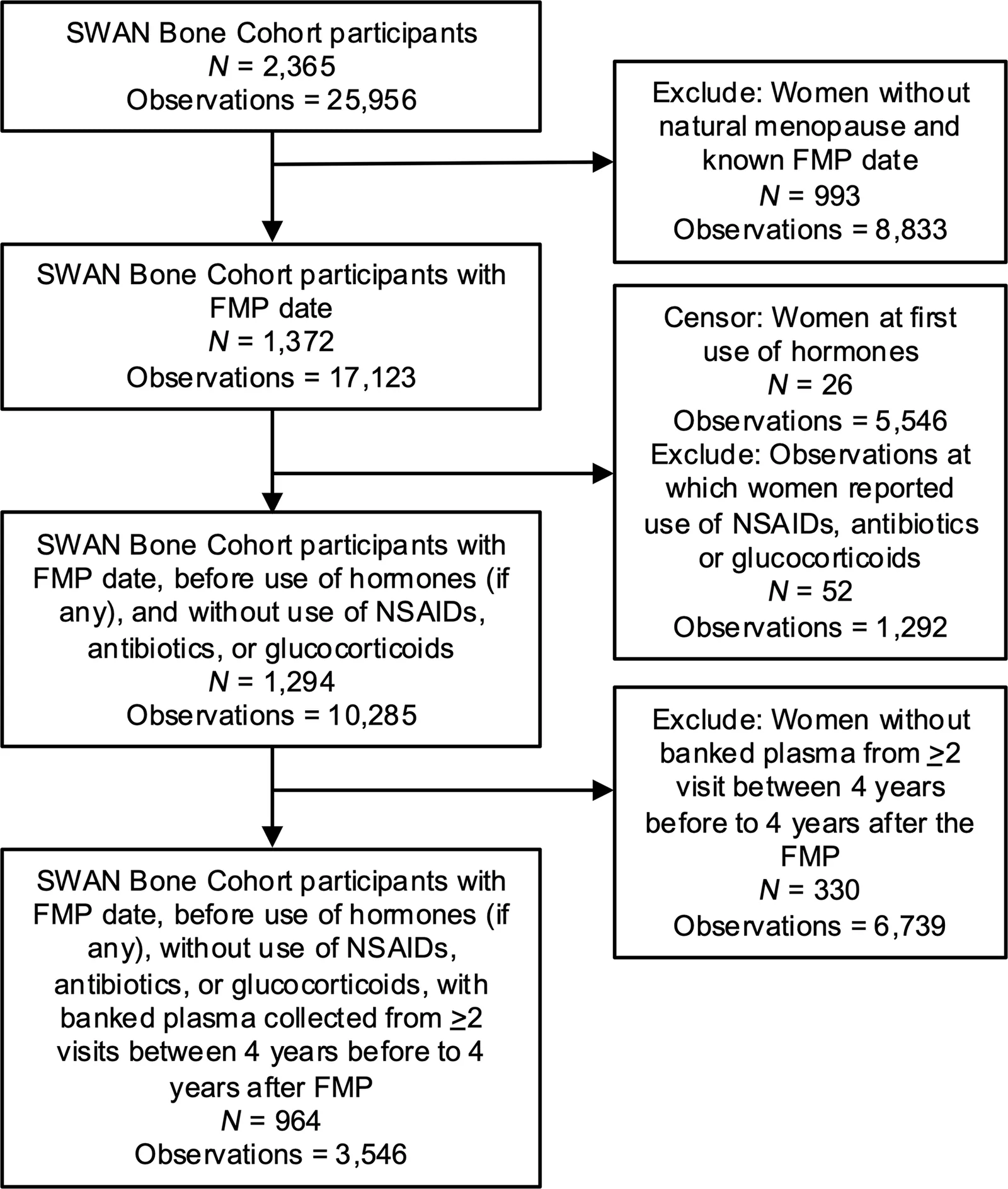
Derivation of study sample. FMP, final menstrual period.

The current study sample was derived from the SWAN Bone Cohort, a subset of 2,365 participants from 5 study sites (exclusive of Chicago and Newark). Starting with the Bone Cohort, we excluded participants who did not have a natural menopause and known FMP date, censored participants at first reported use of sex steroid hormones, and excluded study visits at which women reported using medications that could affect gut permeability or inflammation (NSAIDs, glucocorticoids, and antibiotics). From the remaining participants, we identified those who had banked plasma from ≥2 study visits during the interval spanning 4 years before to 4 years after the FMP (the period when we expected FABP2 and sCD14 to increase most rapidly). The resulting study sample consisted of 964 women, among whom we obtained 3,546 serial assessments of FABP2 and sCD14 (Figure 5).

### Outcomes.

Outcomes were indicators of compromised gut epithelial barrier integrity, operationally defined as (a) gut barrier dysfunction (assessed by FABP2) and (b) translocation of gut microbial products (estimated using sCD14). FABP2 and sCD14 were quantified from stored plasma samples. Based on sample availability, we assayed FABP2 and sCD14 at least 2 and up to 7 times per participant, as follows: (a) at least 2 and up to 5 times during the interval spanning 4 years before to 4 years after the FMP, (b) at the first (earliest) study visit that occurred prior to 4 years before the FMP (if sample was available), and (c) the last (latest) study visit that occurred subsequent to 4 years after FMP (if sample was available). Stored plasma samples from the SWAN baseline visit are reserved and were, therefore, not available to the current project. We refer to the first instance at which we assayed FABP2 and sCD14 as the initial analysis visit.

All FABP2 and sCD14 assays were performed at the UCLA AIDS Institute. FABP2 was quantified using the Quantikine Human FABP2/I-FABP ELISA (R&D Systems; MAB30781, RRID:AB_1964575). sCD14 was assayed using the Luminex xMAP platform with custom-made panels (R&D Systems; DC140, RRID:AB_3095885). Assays for the same woman were performed in a single batch. Intra-assay coefficients of variation were <2.9% for both FABP2 and sCD14.

### Primary predictor.

The primary independent variable was number of years before or after the FMP that FABP2 or sCD14 was measured. The FMP date was defined as the last reported occurrence of menstrual bleeding before a woman was classified as postmenopausal (≥12 consecutive months of amenorrhea). We calculated years from FMP using the FMP date and the collection dates of plasma for FABP2 and sCD14 assessments.

### Covariates.

Covariates included self-reported race and ethnicity (Black, Chinese, Japanese, or White), BMI, and age at FMP. SWAN measured body weight and height using calibrated scales and stadiometers. We calculated BMI as weight in kilograms divided by the square of height in meters.

### Statistics.

Using means (SD) for normally distributed continuous variables, medians (p25, p75) for continuous variables with skewed distributions, and counts (percentages) for categorical variables, we summarized the characteristics of the current study sample and the SWAN Bone Cohort. For ease of interpretation, we natural log-transformed FABP2 (ln[FABP2]) and sCD14 (ln[sCD14]) values for analyses.

We characterized the separate trajectories of FABP2 and sCD14 relative to years from the FMP using a 3-step approach (24). In step 1, we examined the shape of the FABP2 and sCD14 trajectories from pre- to postmenopause using LOESS plots of ln[FABP2] or ln[sCD14] as functions of years before or after the FMP. These plots indicated that trajectories of both markers were piecewise linear, with 3 linear segments and 2 inflection points where the ln[FABP2] and ln[sCD14] slopes changed. The inflection points for ln[sCD14] trailed those by ln[FABP2] by 0.5 years (FMP –2.5 years and FMP +6 years for ln[FABP2]; FMP –2 years and FMP +6.5 years for ln[sCD14]).

In step 2, we formally tested whether the slopes of ln[FABP2] or ln[sCD14] were significantly different before and after each candidate inflection point identified in step 1. Specifically, we employed mixed effects linear regression (no covariate adjustment) to fit piecewise-linear growth curves to repeated measures of each marker of compromised gut epithelial integrity as functions of years from FMP, using linear splines with knots at FMP –2.5 and +6 years for ln[FABP2] and knots at FMP –2 years and FMP +6.5 years for ln[sCD14]. We included random effects for the intercept and 3 slopes to account for within-woman correlations. For both ln[FABP2] and ln[sCD14], slope changes were statistically significant at each of the 2 candidate knots (*P* values for all 4 slope changes < 0.0001). We tested the accuracy of knot selection by varying knot placement in 6-month intervals around each candidate inflection point to examine whether the candidate knot offered the largest explained proportion of within-woman variance (pseudo R-square) (24).

Upon confirming statistically significant slope differences between successive linear segments, step 3 quantified annualized rates of ln[FABP2] and ln[sCD14] change during each piecewise segment and examined whether race and ethnicity, BMI, or age at FMP affected ln[FABP2] or ln[sCD14] change rates. To the mixed effects linear regression models derived from step 2, we added, as fixed effects on the intercept and 3 slopes, race and ethnicity, BMI at the initial visit, age at FMP, and SWAN study site. We then multiplied ln[FABP2] or ln[sCD14] slopes and 95% confidence intervals by 100 to allow interpretation as percentage change in raw FAPB2 or sCD14 per year.

When constructing models, we stipulated the referent participant as White with overall sample mean values for age at FMP (52.16 years) and BMI (27.26 kg/m^2^). Thus, we first report annualized changes in FABP2 and sCD14 for the referent individual, followed by how these slopes vary by race and ethnicity, age at FMP, or BMI.

To examine whether gut barrier dysfunction (FABP2) was a pathway for gut microbial product translocation (sCD14), we employed 2 steps. First, we assessed the longitudinal associations of FABP2 with sCD14 using IFE regression. IFE regression strengthens causal inference in observational studies by relying on multiple repeated measures to quantify the associations of within-woman changes in independent (FABP2) and dependent (sCD14) variables, while eliminating all confounding by the participants’ time-fixed characteristics (e.g., race and ethnicity, genetics) (66–68). To control for time-varying variables, we adjusted for chronological age and BMI (values obtained from the same study visits at which samples for FABP2 and sCD14 measurements were collected). To further explore whether FABP2 was in the pathway of time from FMP to sCD14, we assessed whether adding FABP2 to the model used to generate the sCD14 slopes (described above) attenuated the association of years from FMP with sCD14.

For all analyses, a *P* value less than 0.05 was considered statistically significant.

### Study approval.

Participants provided written informed consent, and each site obtained institutional review board approval.

### Data availability.

Parent SWAN data from study visits through follow-up visit 10 are available at the National Archive of Computerized Data on Aging (https://www.icpsr.umich.edu/web/ICPSR/series/00253). SWAN data from all study visits through follow-up visit 16 are available through National Institute on Aging (NIA) Biobank (https://agingresearchbiobank.nia.nih.gov/studies/swan). FABP2 and sCD14 data were not obtained as part of the parent SWAN study; these data are available by reasonable request to the corresponding author. Values for data points used to generate Figures 1–4 and Tables 1 and 2 are reported in the Supporting Data Values file; supplemental material available online with this article; https://doi.org/10.1172/JCI205059DS1

## Author contributions

AS contributed to study design, statistical analysis, and primary manuscript drafting. ME contributed to performance of assays for markers of compromised gut epithelial barrier integrity and critical review of manuscript. ASK contributed to statistical analysis and critical review of manuscript. RJ contributed to study design and critical review of manuscript. RP contributed to study design and critical review of manuscript. GAG contributed to participant recruitment for the parent SWAN study, study design, and primary manuscript drafting.

## Conflict of interest

The authors have declared that no conflict of interest exists.

## Funding support

This work is the result of NIH funding, in whole or in part, and is subject to the NIH Public Access Policy. Through acceptance of this federal funding, the NIH has been given a right to make the work publicly available in PubMed Central.

- NIH, DHHS, through the National Institute on Aging (NIA), the National Institute of Nursing Research (NINR), and the NIH Office of Research on Women’s Health (ORWH) (Grants U01NR004061; U01AG012505, U01AG012535, U01AG012531, U01AG012539, U01AG012546, U01AG012553, U01AG012554, U01AG012495, and U19AG063720; to SWAN).
- NIH 5R01AR081794; principal investigator: AS.
- NIH, National Institute of Arthritis and Musculoskeletal and Skin Diseases (Grant R01AR075729).

## Supplementary Material

ICMJE disclosure forms

Supporting data values

## Figures and Tables

**Table 4:**
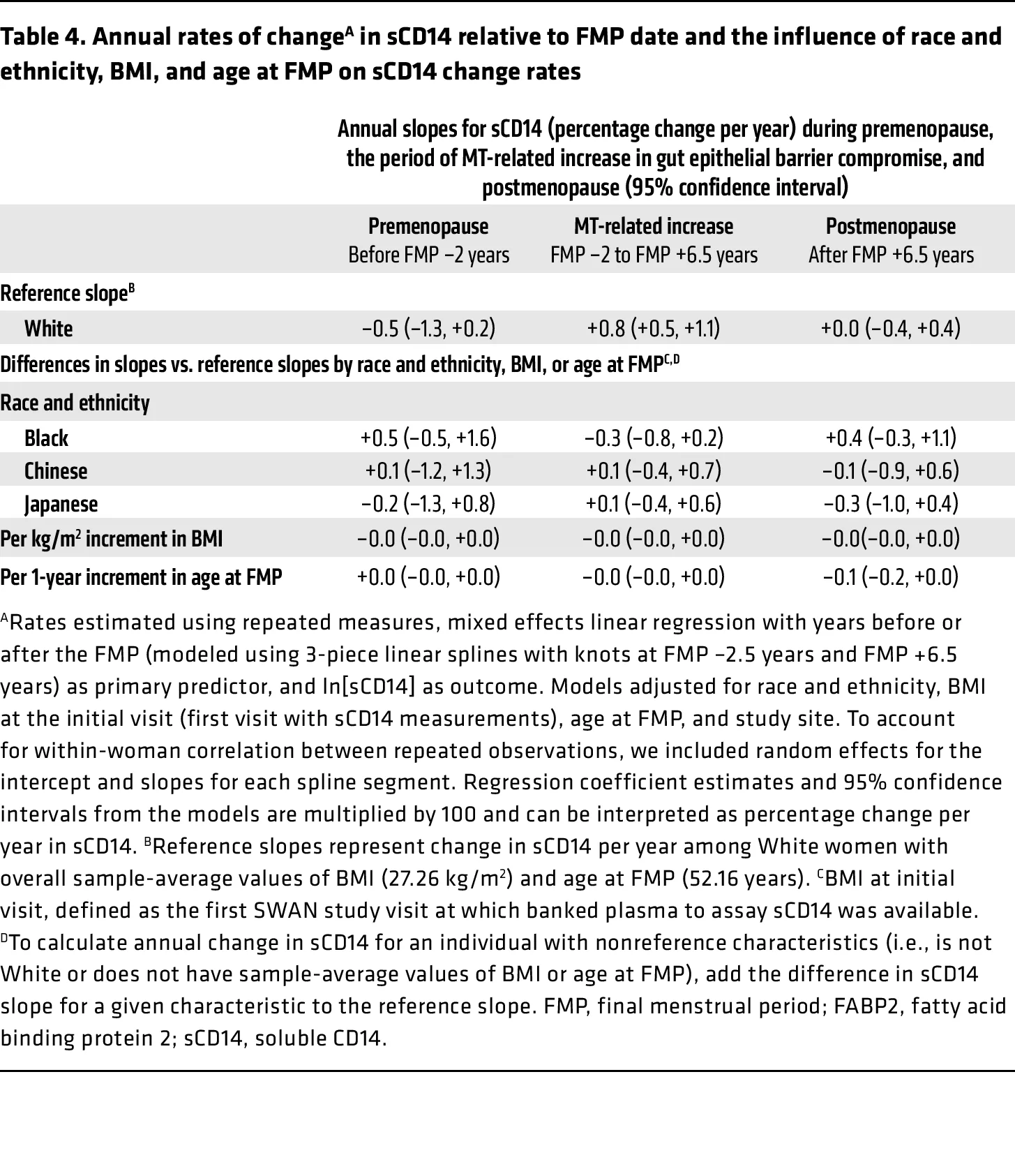
Annual rates of change^A^ in sCD14 relative to FMP date and the influence of race and ethnicity, BMI, and age at FMP on sCD14 change rates

**Table 3:**
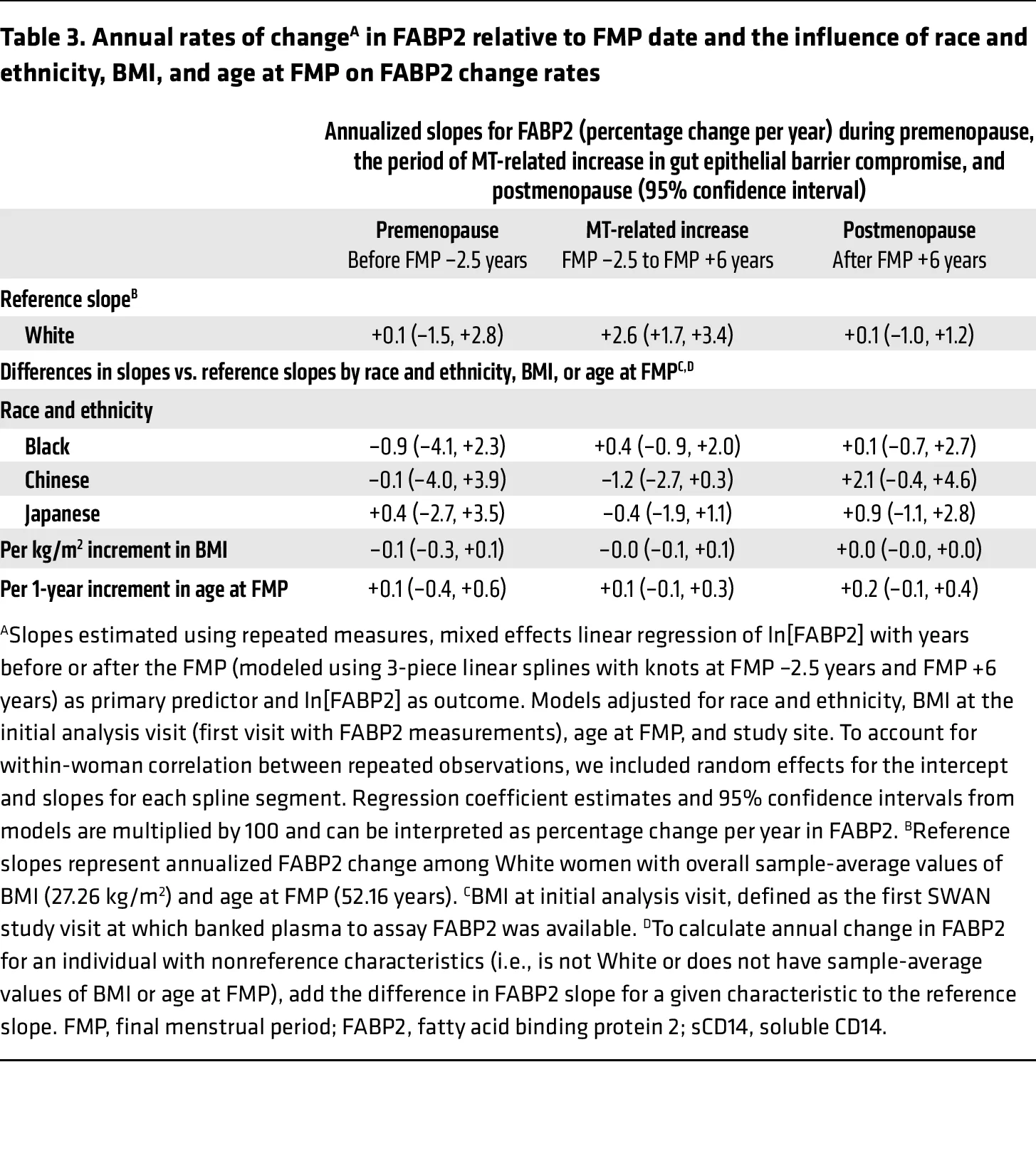
Annual rates of change^A^ in FABP2 relative to FMP date and the influence of race and ethnicity, BMI, and age at FMP on FABP2 change rates

**Table 2:**
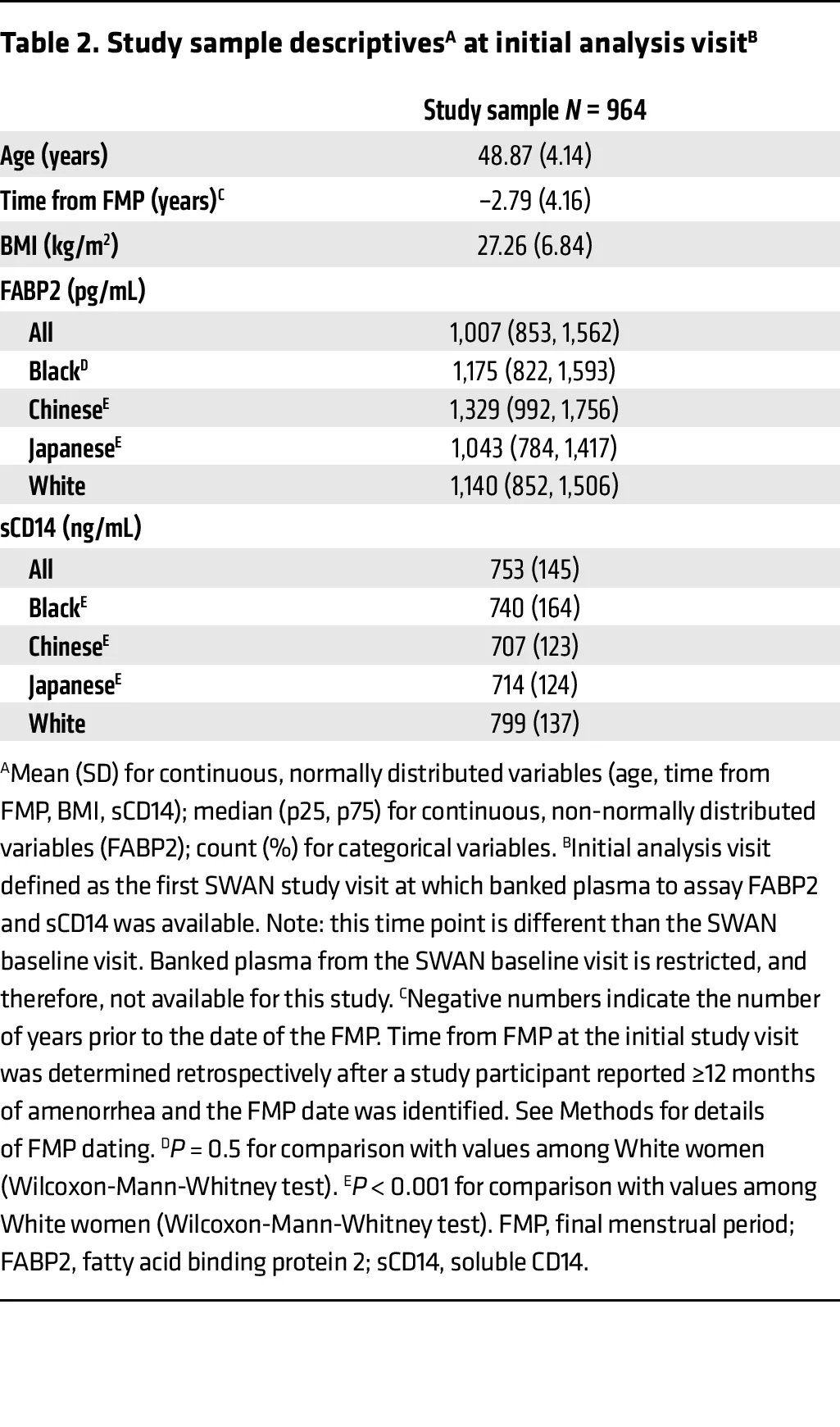
Study sample descriptives^A^ at initial analysis visit^B^

**Table 1:**
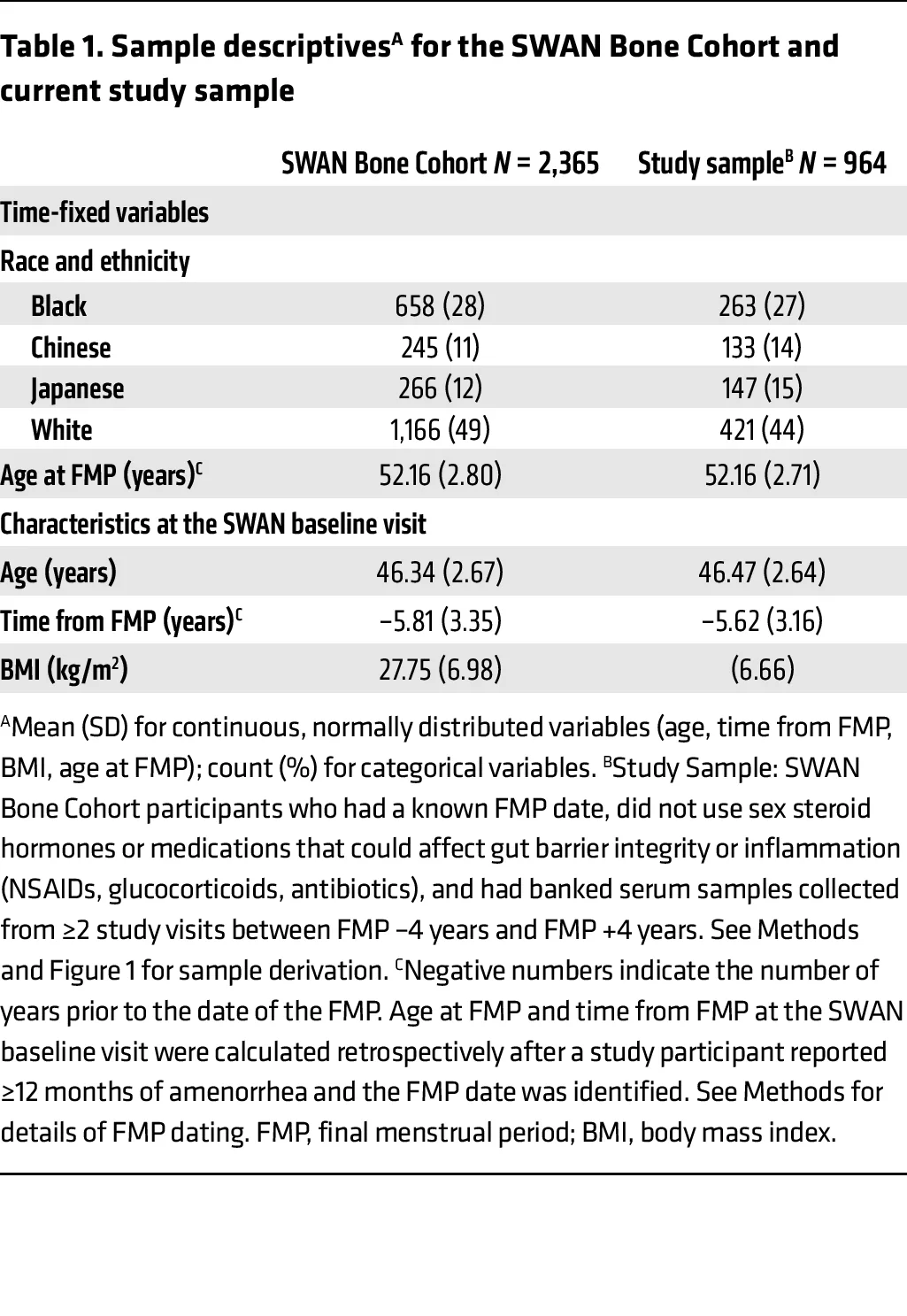
Sample descriptives^A^ for the SWAN Bone Cohort and current study sample

## Version 1 07/09/2026

In-Press Preview

## Version 2 09/01/2026

Electronic publication
