# Corrigendum to Markers of compromised gut epithelial barrier integrity increase during the menopause transition

**DOI:** 10.1172/JCI213400

**Published:** 2026-11-02

**Authors:** Albert Shieh, Marta Epeldegui, Arun S. Karlamangla, Rheinallt Jones, Roberto Pacifici, Gail A. Greendale

Original citation: *J Clin Invest*. 2026;136(17):e205059. https://doi.org/10.1172/JCI205059.

Citation for this corrigendum: *J Clin Invest*. 2026;136(21):e213400. https://doi.org/10.1172/JCI213400.

The results section has been updated to correct the upper limit of the confidence interval for the annual reported increase for FABP2. The correct text appears below.

During its period of MT-related increase (FMP –2.5 to FMP +6 years), FABP2 rose by 2.6% per year (95% CI 1.7 to 3.4%; *P* < 0.0001).

The HTML and PDF versions of the text have been updated online.

The authors regret the error.

## Version 1 11/02/2026

Electronic publication

